# Design and Functional Testing of a Multichamber Perfusion Platform for Three-Dimensional Scaffolds

**DOI:** 10.1155/2013/123974

**Published:** 2013-12-23

**Authors:** Marco Piola, Monica Soncini, Marco Cantini, Nasser Sadr, Giulio Ferrario, Gianfranco B. Fiore

**Affiliations:** ^1^Politecnico di Milano, Dipartimento di Elettronica, Informazione e Bioingegneria, P.zza Leonardo da Vinci 32, 20133 Milano, Italy; ^2^Universitat Politècnica de València, Center for Biomaterials and Tissue Engineering, Camino de Vera, s/n 46022 Valencia, Spain; ^3^Cell and Tissue Engineering Laboratory, IRCCS Istituto Ortopedico Galeazzi, via R. Galeazzi 4, 20161 Milano, Italy; ^4^Dipartimento di Scienze Cliniche L. Sacco, Università di Milano, via G.B. Grassi 74, 20157 Milano, Italy

## Abstract

Perfusion culture systems are widely used in tissue engineering applications for enhancing cell culture viability in the core of three-dimensional scaffolds. In this work, we present a multichamber confined-flow perfusion system, designed to provide a straightforward platform for three-dimensional dynamic cell cultures. The device comprises 6 culture chambers allowing independent and simultaneous experiments in controlled conditions. Each chamber consists of three parts: a housing, a deformable scaffold-holder cartridge, and a 7 mL reservoir, which couples water-tightly with the housing compressing the cartridge. Short-term dynamic cell seeding experiments were carried out with MC3T3-E1 cells seeded into polycaprolactone porous scaffolds. Preliminary results revealed that the application of flow perfusion through the scaffold favored the penetration of the cells to its interior, producing a more homogeneous distribution of cells with respect to dropwise or injection seeding methods. The culture chamber layout was conceived with the aim of simplifying the user operations under laminar flow hood and minimizing the risks for contamination during handling and operation. Furthermore, a compact size, a small number of components, and the use of bayonet couplings ensured a simple, fast, and sterility-promoting assembling. Finally, preliminary *in vitro* tests proved the efficacy of the system in enhancing cell seeding efficiency, opening the way for further studies addressing long-term scaffold colonization.

## 1. Introduction

Many efforts have been made to design proper solutions for the development of culture systems to support cell culture in 3D porous matrices. Different experimental set-ups and approaches have been proposed, spanning from static to dynamic culture conditions [1]. Traditional static culture systems, such as multiwell plates or Petri dishes, have been routinely used in cell culture laboratories due to their ease of use, disposability, sterility-prone handling, and low cost [2]. Nonetheless, these systems suffer from serious limitations in supporting cell culture in 3D scaffolds. Indeed, diffusion-based mass transport within 3D constructs is limited, and this causes critical concentration gradients [3] (pH, dissolved oxygen, metabolites, and glucose), poor control on the culture milieu, and lack of cell stimulation. These limitations drastically reduce the thickness of the sample that can be cultured in such static systems (more or less 1-2 mm), prompting the developmentof *ad hoc* dynamic culture bioreactors [1]. Several solutions, including stirred and spinner flasks [4, 5], rotating wall vessel [6, 7], and perfusion [8–10] bioreactors, were proposed over the years for supporting cell growing or tissue maturation. While stirred and rotating wall vessel bioreactors are able to create an environment where the nutrients are homogeneously distributed only on the external surface of the scaffolds, perfusion bioreactors were proposed to enhance the mass transport in the inner core of porous scaffolds and to provide a controllable culture environment throughout their thickness [1, 11]. Confined perfusion bioreactors [3, 12–14] ensure a proper nutrient supply/waste removal to virtually all the cells involved in the culture, enhancing mass transport phenomena, and avoid unwanted concentration gradients of oxygen [15], nutrients, pH, and soluble growth factors by forcing the medium to flow through the scaffold-pore network [1]. Flow perfusion bioreactors increase the quality, reproducibility, and uniformity of seeding procedures, compared to the dropwise, injection, or cell-suspension seeding methods that are adopted in static cultures [8, 9, 16]. In addition, these bioreactors may be used to induce controlled biomechanical stimuli to the cells, mainly shear stress [17], representing appealing tools to replicate the natural conditions of different tissues such as bone [18, 19], cartilage [20, 21], and liver [22].

Nevertheless, state-of-the-art perfusion bioreactors suffer from various limitations. A crucial limitation is related to cumbersome handling and assembling procedures: O-ring couplings or screws-and-nuts fixations [10, 14, 18, 23] are unpractical for the common laboratory operator, and they increase the time required for device assembly and the risk for sterility loss. Most of the perfusion bioreactors reported in the literature are complex systems, with high priming volume [12, 14], not suitable for testing different culture conditions simultaneously while independently avoiding cross-contamination [14, 18].

In this work, we developed and validated a multichamber perfusion system, compatible with the standards for laboratory safety, providing a straightforward, user-friendly platform for dynamic cell cultures in confined-perfusion conditions. The design of the bioreactor aimed at combining the well-established advantages of confined perfusion with the benefits of conventional static culture systems such as multiwell plates or culture flasks (safety, compactness, ease of use, and low cost).

## 2. Materials and Methods

### 2.1. Confined Flow Perfusion Platform

#### 2.1.1. Design Specifications

Bioreactor design took into account the general specifications of a cell culture system [1], with particular attention dedicated to the ease of assembly under laminar flow hood and the safety of use in a cell culture laboratory. In particular, the following basic requirements were addressed:

- biocompatibility of materials, plus their stability in time, in the perspective of long-term cultures and/or multiple uses;
- easy processability of materials and suitability for surface polishing;
- transparency, to ensure visual inspection for air bubble detection and/or medium color checking;
- compatibility with sterilization processes, for example, via autoclaving and/or hydrogen peroxide plasma;
- suitability for system scaling to the independent and simultaneous testing of different culture conditions.

In addition, confined-flow perfusion was made independent of the scaffold size and shape (within certain limits) by designing interchangeable cartridges devoted to holding the scaffold in place while ensuring a tight seal between the scaffold and the culture chamber and avoiding nonperfusing medium flow.

Specific measures were then taken to make the system operator-friendly and prone to sterility preservation. This was pursued (i) by minimizing the number of parts composing the bioreactor and (ii) by making the device overall size as compact as required for the operator maneuvers to resemble those of conventional static culture. Minimization of priming volume was also sought for, to limit the cost of soluble culture medium compounds and to enable medium exchange by means of standard pipetting techniques.

#### 2.1.2. Bioreactor Design

**Figure 1:**
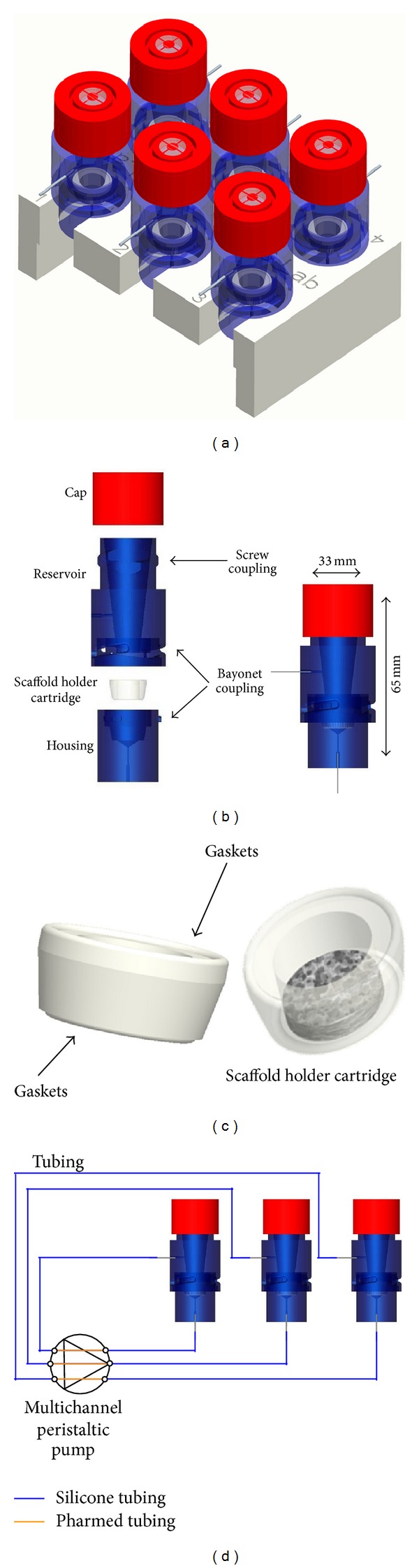
CAD model of the six-chamber bioreactor (a). CAD model of the single culture chamber: exploded and assembled view; the culture chamber is composed by a reservoir, a disposable screw cap, a housing, and a scaffold holder cartridge (b). Detail of a scaffold holder cartridge with a 10 mm opening diameter; the arrows indicate the upper and lower gaskets; the cartridge allows hosting matrices with different sizes and shapes (c). Sketch of the fluidic circuit with parallel culture chambers and perfusion circuits, actuated by a multichannel peristaltic pump (three out of six parallel circuits/chambers are shown) (d).

A modular multichamber dynamic culture system was designed by means of the 3D CAD software Pro/Engineer Wildfire 4.0 (PTC, Needham, MA). In particular, the device comprises 6 independent culture chambers with independent fluidic circuits (Figure 1(a)).

Each chamber consists of three custom-made parts (Figure 1(b)): (i) a deformable scaffold holder cartridge, (ii) a rigid housing, and (iii) a reservoir element (up to 7 mL-volume), plus a commercial disposable vented screw cap.

The scaffold holder cartridge (Figure 1(c)) acts as an interface between the scaffold and the housing, ensuring the medium to be forced through the core of the scaffold. In addition, it is provided with integrated gasket profiles, which constitute the sealing elements between the housing and the reservoir. It was manufactured with a deformable and biocompatible silicone elastomer (Sylgard 184, Dow Corning Corporation, Midland, MI) and prepared by injection molding/vacuum casting and curing within purposely manufactured polymethylmethacrylate (PMMA, Plasting s.r.l., Segrate, Italy) molds. The scaffold holder cartridge hosts scaffolds with a diameter up to 10 mm and a thickness up to 6 mm. The housing and the reservoir were manufactured by computer numerical control machining from PMMA blocks. The inner surface profiles are free of sharp angles or slits and were polished until translucent. The reservoir is mounted on top of the housing by an integrated 1/4-turn bayonet coupling. Two stainless steel connectors (AISI 304, MDL s.r.l, Sondrio, Italy) glued at the base of the housing and at the side surface of the reservoir (Araldite 2011 AW106/HV953U, two-part glue, Vantico s.r.l., Varese, Italy) serve as tubing connection sites. A standard commercial vented cap (Sarstedt AG & Co, Numbrecht, Germany) covers the reservoir, separating the culture environment from the external environment for sterility maintenance; meanwhile, it guarantees gas exchange through the free surface of the medium contained in the reservoir.

Once assembled, the culture chamber is hosted in a support made of polyoxymethylene, which holds 6 culture chambers in the planar size of a standard multiwell plate (130 mm × 85 mm).

The fluidic circuit (Figure 1(d)) is composed of autoclavable oxygen-permeable silicone tubing with an inner diameter of 0.8 mm and a wall thickness of 0.8 mm (Platinum Cured Silicone, Cole Parmer, Vernon Hills, IL) and of autoclavable polypropylene-based pump tubing with internal diameter of 0.63 mm and wall thickness of 0.9 mm (Pharmed BPT Pump Tubing, Carlo Erba Reagenti, Milano, Italy). Polypropylene autoclavable luer connectors (Cole Parmer, Vernon Hills, IL) were used to guarantee leak-free connections and facilitate circuit assembly under a laminar flow hood. A multichannel peristaltic pump (IPC-N, Ismatec SA Labortechnik-Analytik, Glattbrugg, Switzerland) draws culture medium from the reservoir towards the housing (hence through the scaffold) or *vice versa*, with a flow rate spanning from 0.4 *μ*L/min to 11 mL/min, depending on the pump tubing size (inner diameter from 0.13 mm to 3.17 mm).

The bioreactor system was designed to work within a cell culture incubator, and the control of the milieu parameters, such as temperature, gas composition (O_2_ and CO_2_), and relative humidity, is guaranteed by the incubator itself. Gas exchanges between the culture chamber and the incubator environment occur through (i) the free surface of the medium in the integrated reservoir, facilitated by the cap provided with HEPA filter and (ii) the oxygen-permeable silicone tubing wall, used as an oxygenator element. The silicone tubing length was sized according to the literature to guarantee appropriate oxygenation [24]. Assuming an oxygen consumption rate equal to 3 × 10^−8^ mmol/s (i.e., cellular uptake of 1.25 × 10^−17^ mol s^−1^/cell [25] and total cell density of 10^7^ cell/mL) for a 3 mm thick, 10 mm diameter scaffold and considering the silicone tube size, the tubes were sized at least 50 cm long.

### 2.2. Functional Tests

Since possible leakages of culture medium would compromise the sterility and the functionality of the device, the bioreactor system was firstly bench-tested to check water tightness under working conditions, applying flow rates up to 11 mL/min. After this preliminary test, we thoroughly investigated the sterility maintenance of the device, which was followed by cell seeding tests in polycaprolactone (PCL) scaffolds.

#### 2.2.1. Sterility Maintenance Test

Considering the relevance of the sterility maintenance over time, a systematic approach was used for its verification over several days. The adopted protocol consisted of a first step of cleaning and disinfection of the components with a detergent and 70% ethanol (for the tubing, connectors, and scaffold holder cartridge) or with a mixture of deionized water and sulfuric acid (for the components of the culture chamber in PMMA), to abate the initial bacterial load. Then, the fluidic circuit and the scaffold holder cartridge were autoclaved for 30 minutes at 121°C (Europa B XP 24, Tecno-Gaz, Parma, Italy), while the housing and reservoir were sterilized via hydrogen peroxide plasma (Sterrad System, ASP, Irvine, CA). The medium used to fill the circuit consisted of Dulbecco's Modified Eagle Medium (DMEM, Sigma Aldrich Corporation, MO) supplemented with 100 U/mL penicillin (EuroClone, Italy), 0.1 mg/mL streptomycin (EuroClone, Italy), 10% fetal bovine serum (EuroClone, Italy), and 2 Mm L-glutamine (EuroClone, Italy).

**Figure 2:**
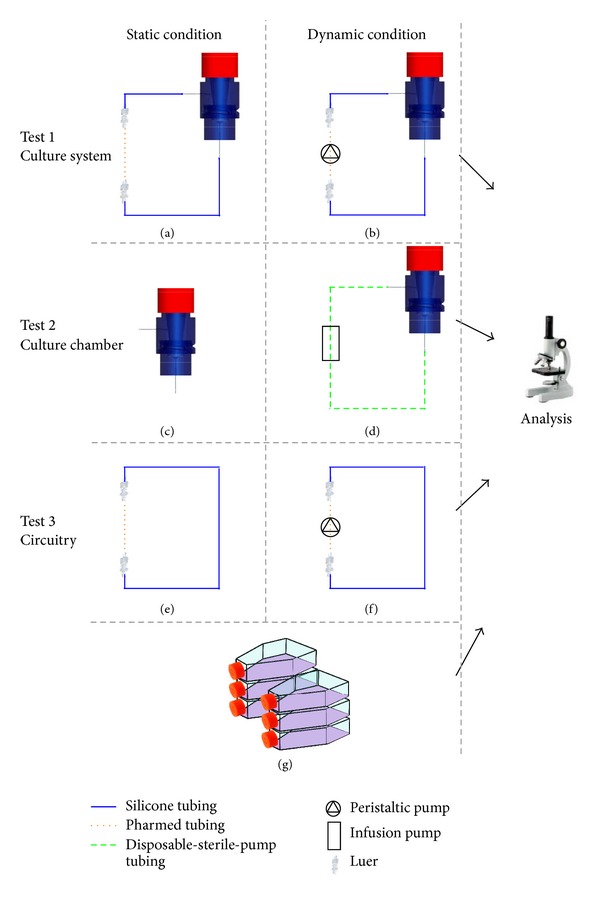
Static (a, c, e) and dynamic (b, d, f) tests performed for evaluating the bioreactor sterility maintenance. Test 1: evaluation of the culture system (Ismatec multichannel peristaltic pump, fluidic circuit and culture chamber) (a, b). Test 2: evaluation of the single culture chamber (c) with a disposable-sterile-silicone-pump tubing Infusomat Standard (BBraun) and fed in the infusion pump Infusomat fmS (d). Test 3: evaluation of the preassembled circuitry alone in static (e) and dynamic (f) condition, where an Ismatec peristaltic pump is used. As a control, a T-flask containing the same medium, used for all tests, was incubated (g).

Maintenance of sterility was tested without use of the scaffold and for a period of 14 days, with medium replacement every 3 days, reproducing standard culture conditions, applying a flow rate of 3 mL/min. Static and dynamic tests were performed in triplicate in different testing configurations. The whole culture system's sterility was investigated in the full configuration (culture chamber connected with the disposable circuitry and fed with an Ismatec peristaltic pump: Figure 2, tests 1(a) and 1(b)), whereas the sterility of the culture chamber alone was investigated by coupling the chamber with a disposable sterile silicone pump tubing (Infusomat Standard, BBraun, Melsungen, DE) and infusion pump (Infusomat fmS, BBraun) (Figure 2, tests 2(c) and 2(d)). The sterility of the disposable circuitry alone was also investigated by looping it and using the Ismatec peristaltic pump (Figure 2, tests 3(e) and 3(f)). As a control, a T-flask containing the same medium, used for all the tests, was also incubated (Figure 2(g)). All the aforementioned set-ups were maintained in a cell culture incubator (CO_2_ 5% and 37°C). A schematic representation of the sterility experiments is shown in Figure 2.

To check for contamination, the culture medium, collected from each trial, was analyzed via inverted microscopy, and bacterial growth was evaluated on solid and liquid microbiological mediums. For the bacterial growth evaluation, the culture medium, collected from the various tests, was centrifuged, and the pellet and part of the supernatant were inoculated into the growth medium and incubated. To assess the total bacterial growth, the Plate Count Agar solid medium (Biolife Italiana s.r.l., Milan, Italy) was used and the sample was incubated for 72 hours at 30°C; the Chocolate Agar + PolyViteX solid medium (bioMérieux SA, Marcy l'Etoile, France) and the Brain Heart Infusion broth were used to assess the proliferation of microaerophilic bacteria by incubating the sample in microaerophilic conditions (5% O_2_, 10% CO_2_, 85% N_2_, at 40°C).

#### 2.2.2. Cell Seeding Experiments within Scaffolds

A preliminary experiment of dynamic cell seeding was carried out with the aim of verifying the performance of the device. In particular, the functionality of the culture system was investigated using the bioreactor for the dynamic seeding of the mouse preosteoblastic clonal cell line MC3T3-E1, obtained from the RIKEN Cell Bank (RIKEN BioResource Center, Tsukuba, Japan). Prior to seeding, cells were maintained in DMEM (Thermo Fisher Scientific, Inc., Waltham, MA) supplemented with 10% fetal bovine serum (FBS) (Thermo Fisher Scientific, Inc., Waltham, MA), 100 U/mL penicillin (EuroClone, Italy), and 0.1 mg/mL streptomycin (EuroClone, Italy) and passaged twice a week using standard techniques. For the seeding experiments, a cell seeding density of 2 × 10^5^ cells/sample in 50 *μ*L medium was chosen based on the literature data for both dynamic and static conditions [16].

Cylindrical PCL porous scaffolds (5.5 mm diameter, 2 mm thick, with a porosity of 84%), manufactured via a compression molding/particulate leaching technique, were used, after sterilization and preconditioning with culture medium. Briefly, scaffolds were sterilized by ethanol 70% and then preconditioned with culture medium at 37°C and 5% CO_2_ overnight.

For the dynamic cell seeding, three flow rates were applied: 0.03 mL/min, 0.1 mL/min, and 0.3 mL/min, corresponding to superficial velocities, at the scaffold impact surface of 21 *μ*m/s, 70 *μ*m/s, and 210 *μ*m/s, respectively. Cell suspensions were poured onto the top surface of the scaffolds, with each scaffold being fixed within a holder cartridge and hosted within a culture chamber; then each chamber was capped with a reservoir, and each reservoir was filled with 7 mL of additional medium and closed. The system was finally incubated (at 37°C and 5% of CO_2_) for 6 hours. Culture media were pumped through the scaffolds in a top-to-bottom direction to favor cell penetration through the scaffold depth. As a static control, suspended cells were seeded dropwise onto or injected into the scaffolds, with each scaffold being fixed within a holder cartridge and hosted in a well of a multiwell plate. Subsequently, each well was gently filled with medium to cover the entire scaffold holder cartridge, and each sample was incubated at 37°C and 5% of CO_2_ for 6 hours. All the cell seeding experiments were repeated thrice. At the end of the culture, all the samples were washed with Dulbecco's Phosphate Buffer Saline (DPBS, Sigma-Aldrich, USA) twice, then fixed with 10% formalin (Sigma-Aldrich, USA) for 1 hour at room temperature, and finally maintained in DPBS at 4°C until characterization.

Formalin-fixed scaffolds were cryoprotected and cut into longitudinal sections of 200 *μ*m thickness, in order to maintain the material continuity, using a cryostat microtome. The sections were then analyzed via fluorescence microscopy (Leica DM6000B, Leica Microsystems GmbH, Wetzlar, Germany) to investigate cell distribution within the scaffold, as a seeding performance readout. Cell nuclei were stained with DAPI (Invitrogen Corp., Carsalbad, CA), according to the manufacturer protocol.

**Figure 3:**
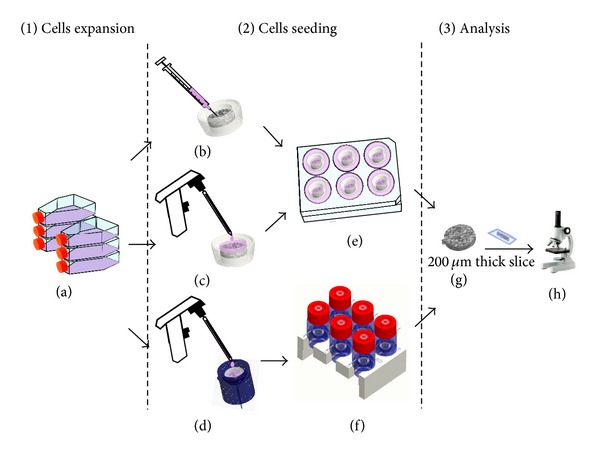
Cell culture experiments performed for the evaluation of the dynamic perfusion seeding. (1) Cell expansion: cell line MC3T3-E1 was cultured in standard T-flask in cell culture incubators (5% CO_2_, 37°C) and passaged when 60–70% confluence was reached (a). (2) Cell seeding: the seeding density was about 2 × 10^5^ cells/50 *μ*L of culture medium, and the scaffolds were porous PCL matrices with a porosity of 84%. Suspended cells were injected (b) or dropwise seeded (c, d) onto the scaffolds. Injection- (b) and dropwise-seeded (c) scaffolds were fixed within holder cartridges and incubated in a six-well plate for 6 hours. In addition, cell suspensions were poured on the top surface of each scaffold fixed in a holder cartridge within a perfusion culture chamber (d); then the bioreactor was closed (f), and incubated for 6 hours. (3) Analysis: formalin-fixed scaffolds were cut into 200 *μ*m thick-longitudinal sections (g), and the distribution of the seeded cells within the thickness of the scaffold was observed via fluorescence microscopy after cell nuclei staining (h).

A schematic representation of the experimental campaign is shown in Figure 3.

## 3. Results 

### 3.1. Bioreactor Design

**Figure 4:**
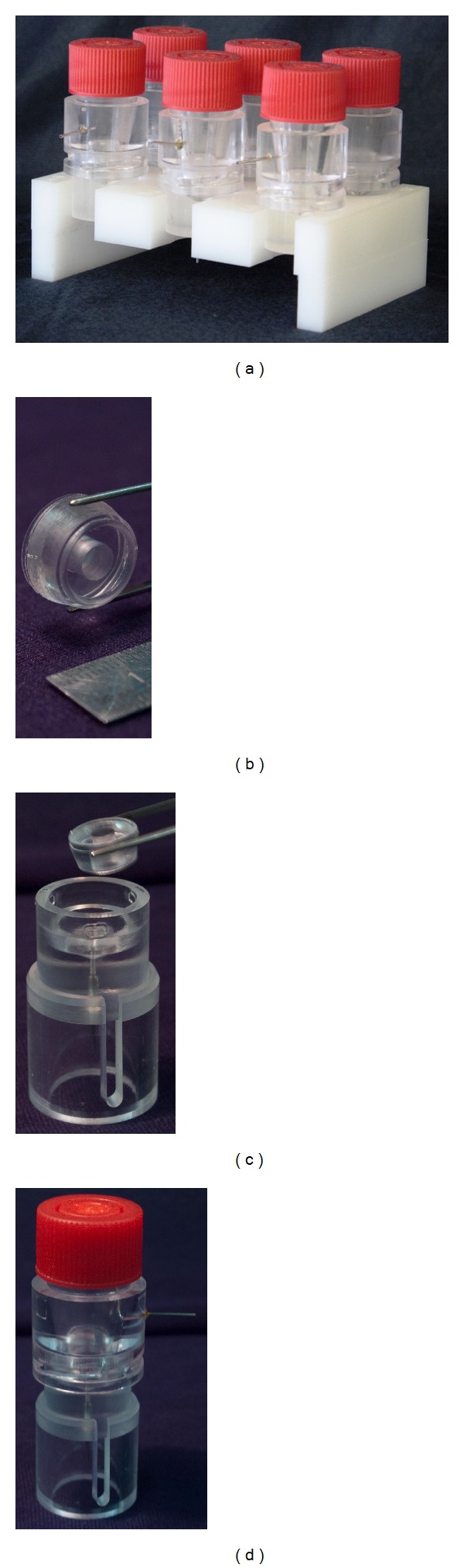
The 6-chamber bioreactor hosted in a support made of polyoxymethylene, with a planar size of a multiwell-plate (a). Detail of a scaffold holder cartridge with a 5.5 mm opening diameter; the upper and lower gaskets can be appreciated (b). The scaffold holder cartridge is fitted into the housing (c), which is topped by a reservoir element through a fast bayonet coupling (d).

The assembled 6-chamber bioreactor is shown in Figure 4(a). A sample of the scaffold holder cartridge is shown in Figure 4(b), where the two integrated gaskets can be appreciated.  Figure 4(c) shows the scaffold holder cartridge insertion into the housing, while a single assembled culture chamber is shown in Figure 4(d).

Hydraulic tests showed no leakage of fluid and proper isolation of the inner chambers from the outside environment. These results demonstrated the reliability and proper functioning of all the designed parts and allowed transferring the bioreactor to a cell culture laboratory for subsequent testing and use.

### 3.2. Sterility Maintenance Experiments

Regarding the sterility tests performed with the culture system, the observation via inverted microscope showed the presence of micron-sized debris in the culture medium collected from all the tests which involved the use of the pump tubing and the peristaltic pump (refer to Figure 2, test 1(b), and test 3(f)). These results suggested that the source of debris was the mechanical stress induced by the rollers of the peristaltic pump onto the pump tubing (spallation phenomenon). Indeed, all the microbiological tests confirmed the absence of any biological contamination either due to yeasts, or aerobic bacteria. Further analyses via the evaluation of growth on solid and liquid microbiological media excluded the presence of anaerobic bacteria in the culture medium.  Table 1 summarizes the results of the sterility maintenance experiments.

### 3.3. Seeding Efficacy Experiments

**Figure 5:**
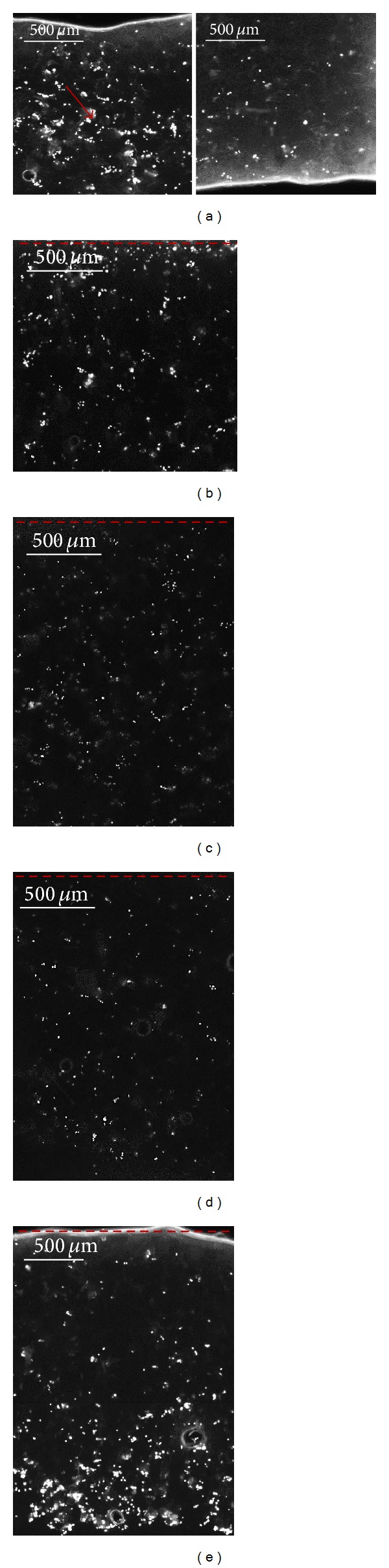
Representative fluorescence microscopy images of MC3T3-E1 cell nuclei stained with DAPI. Longitudinal sections of injection ((a) upper region on the left and lower region on the right), dropwise-seeded scaffolds (b) and perfusion-seeded scaffolds ((c) flow rate 0.03 mL/min, (d) flow rate 0.1 mL/min and (e) flow rate 0.3 mL/min). The red arrow indicates the injection area, and the red dashed lines represents the seeding surfaces.

Cell seeding experiments demonstrated that injection seeding resulted in a nonhomogeneous distribution of the cells within the scaffold (Figure 5(a)). Cells were mainly located in the region of the porous matrix near the injection site, (Figure 5(a), red arrow). In the case of dropwise seeding, most of the seeded cells were retained in the upper part of the matrices (Figure 5(b)) and were not uniformly distributed across the section of the PCL scaffold. In the dynamically seeded scaffolds, the distribution of the cells across the longitudinal section of the scaffold depended on the value of the applied flow rate (Figures 5(c), 5(d), and 5(e)). In particular, at lower flow rates (0.03 mL/min and 0.1 mL/min, Figures 5(c) and 5(d)) cells appeared to be homogenously distributed across the scaffold section and tended to a single-cell distribution, while at higher flow rates (0.3 mL/min, Figure 5(e)) a completely inhomogeneous distribution of the cells was achieved. In this case, the seeded cells were dragged at the bottom end of the scaffold.

## 4. Discussion 

It is well acknowledged that scaffold perfusion in 3D cell cultures may induce an adequate media mixing through the scaffold pores, overcoming the diffusion limitations of static cultures and fostering a more homogeneous culture environment control within the construct [1, 11]. Perfusion bioreactors are now available in many laboratory settings; however, their practical exploitation is often limited by complicated running procedures and by a consequent increase in their contamination potential, compared to conventional static culture systems. In our design, together with basic requisites and perfusion-related functional features, we focused our efforts towards optimizing the user-friendly manipulation of a compact device, thus inherently promoting the device safety. We adopted a confined-flow perfusion scheme and developed a multichamber perfusion platform for seeding and culturing up to 6 scaffolds independently and simultaneously, as in a six-well plate. With the pump system adopted, two identical platforms may be run in parallel, thus reaching a 12-chamber throughput potentiality in the same experiment. This does not compare to the high-throughput capability of other designs [26]. However, by using a completely separated flow channel for each cultured scaffold, we gave priority to avoiding cross-contamination and ensuring strict respectfulness of the imposed flow rate per channel, regardless of possible differences among the hydrodynamics of different scaffolds. This makes our system inherently suitable for screening experiments involving the use of different additives, or one additive at different concentrations, in the flowing medium. Furthermore, scaffold harvesting at different time points, whether necessary, is immediate.

In agreement with our requirements, the design process led to the development of a modular, functional, and safe bioreactor, responding to the best standards for laboratory practice. The design process started with the optimization of a single culture chamber, wherein several practical features were adopted to ensure safety for the culture experiment. This involved minimizing the number of parts that constitute the bioreactor, in order to make a sterile assembly of the device under laminar flow hood simple and fast. In particular, a compact system was conceived, made of three custom-made components (the housing, the cartridge, and the reservoir), and one disposable sterile cap. The small number of components and the device's modularity also made the process of cleansing, disinfection, and decontamination straightforward, enhancing the efficacy of sterilization.

The deformable scaffold holder cartridge permitted an easy handling of the scaffold during assembling, seeding, and harvesting operations under-hood. By simply changing the inner profile of the cartridge, it is possible to host scaffolds with different sizes and shapes within the same housing. The only requirement is for the scaffold to be mechanically resistant to the radial compression that keeps it in place within the cartridge; this makes our system suitable for hosting a wide range of scaffolds: ceramic (e.g., hydroxyapatite porous scaffolds), metallic (e.g., porous titanium or tantalum structures), polymeric (if sufficiently rigid, e.g., polylactic acid, polycaprolactone scaffolds or polyurethane foams), inorganic/polymeric nanocomposites, and so forth. Highly deformable structures such as hydrogels or highly porous polymeric matrices would need a rigid external support hosting them to be kept in place within the cartridge. Resorting to a deformable material for the cartridge proved to be an excellent solution to achieve confined flow perfusion with a single-part scaffold-holding element. This was a valid alternative to the rigid press-fit cassettes reported by Bancroft et al. [13, 27], Janssen et al. [9], and Grayson et al. [18], ensuring a tight seal between the scaffold and the culture chamber and avoiding nonperfusing flow. The 3-thread bayonet coupling, which connects the reservoir to the housing, makes the sterile assembling of the device fast and intuitive for the operator (a 1/4-turn bayonet is more immediate in laboratory use than O-ring couplings or screws-and-nuts fixations [10, 14, 18, 23]) and ensures a homogeneous compression of the deformable scaffold holder cartridge avoiding leakages. The reservoir, after mounting, is fluidically integrated within the culture chamber to reduce the dead priming volume of the overall circuit down to 3.5 mL, quite smaller than the 80 mL reported by Sailon et al. [12] and comparable to the 3 mL stated by Domansky et al. [26]. Priming volume may be increased up to 7 mL by adding medium in the reservoir. This solution guarantees a compact size, minimizes stagnant zones, and limits the costs related to culture reagents. Finally, the employment of the same closing element as that of a T-flask, again a solution that fits common laboratory practice, facilitates under-hood maneuvers such as medium replacement and the addition of soluble factors inside the culture chamber via the reservoir.

Future improvements and refinements are under consideration. The addition of sensors to monitor the culture conditions, for example, pH or partial pressure of dissolved oxygen (pO_2_) in the culture medium, represents the first target. As a matter of fact, monitoring pO_2_ may provide a means to validate the strategy adopted for modeling oxygen transport and consumption within the cultured microenvironment, which was aimed at sizing the length of the tube. A foreseen improvement for the designed system consists in its conversion into the so-called “dynamic multiwell” by integrating a micropumping system within the multichamber device [26]. In addition, an automatic medium exchanging system should be added with the aims of reducing contamination risks due to operator handling and preserving homeostasis.

Consistently with the design guidelines, a systematic approach was used to check the maintenance of sterility in working conditions. The multitude of microbiological techniques adopted to analyze the culture medium collected from each trial allowed excluding the presence of contamination due to aerobic or anaerobic bacteria and yeasts, even if debris in the order of microns, both spherical and needle-shaped, was found. The debris was identified to be due to spallation phenomena induced by the rollers of the commercial peristaltic pump on the pump tubing. Even if in our experience the evidence of spallation debris may have been somewhat amplified by the small overall volume, which caused an increase in debris concentration, the problem of spallation is surprisingly neglected or underestimated in tissue engineering applications [28, 29].

Finally, the seeding experiments gave the proof of the functionality of the bioreactor. The results of the 6-hour cultures of MC3T3-E1 cells revealed that the application of flow perfusion (superficial velocity in the range of 20–70 *μ*m/s) through the scaffold favored the penetration of the cells to its interior. At the same time, flow perfusion at low flow rate allowed cells to be uniformly distributed all over the scaffold section. These results are in agreement with the literature data [8, 16, 30], confirming that 3D culture under confined flow perfusion allows for a better control over the cell fate and behavior by enabling nutrient supply/waste removal, enhancing mass transfer phenomena, and avoiding dangerous concentration gradients of oxygen, nutrients, pH, and soluble growth factors by forcing the medium to flow through the scaffold pore network. In summary, this demonstrated that the multichamber system is a suitable culture platform, beneficially improving cell distribution within the scaffold with respect to standard seeding methods, such as static dropwise or injection seeding.

## 5. Conclusion

A flow perfusion platform was designed, built, and tested. The system is a multichamber bioreactor that is able to host several scaffolds at the same time, allowing the simultaneous and independent investigation of different culture conditions without inducing too much complication in its use with respect to standard static culture systems. Thanks to its versatility, in perspective the bioreactor can be used for different applications, such as bone, cartilage, and liver tissue engineering, and for screening the effects of different culture conditions on cell behavior.

## Figures and Tables

**Table 1:** Data related to microbiological analyses performed to check for contamination of the culture medium collected from each sterility maintenance experiment Neg.: negative result (i.e., zero colonies found). For the test configurations refer to Figure 2.

Test		Plate Count Agar solid medium	Chocolate Agar + PolyViteX solid medium	Brain Heart Infusion broth
1	(a)	Neg.	Neg.	Neg.
	(b)	Neg.	Neg.	Neg.

2	(c)	Neg.	Neg.	Neg.
	(d)	Neg.	Neg.	Neg.

3	(e)	Neg.	Neg.	Neg.
	(f)	Neg.	Neg.	Neg.
